# A Novel Four Single-Sideband M-QAM Modulation Scheme Using a Shadow Equalizer for MIMO System Toward 5G Communications †

**DOI:** 10.3390/s19081944

**Published:** 2019-04-25

**Authors:** Mohammed Mustafa Alhasani, Quang Ngoc Nguyen, Gen-Ichiro Ohta, Takuro Sato

**Affiliations:** 1 Department of Communications and Computer Engineering, Faculty of Science and Engineering, Waseda University, Shinjuku-ku, Tokyo 169-0051, Japan; t-sato@waseda.jp; 2 Yokosuka Telecom Research Park, 3-4 Hikarinooka, Yokosuka, Kanagawa 239-0847, Japan; ohtagenichiro859@gmail.com

**Keywords:** four single-sideband (4-SSB), the guard interval discrete Fourier transform spread orthogonal frequency division multiplexing (GI DFT-s-OFDM), M-ary quadrature modulation amplitude 4-SSB (M-QAM 4-SSB), shadow equalizer, SISO equalizer, massive multiple-input multiple-output (m-MIMO), Hilbert transformation, 5G communications

## Abstract

Single-sideband (SSB) modulation through Hilbert transformation has successfully transmitted data using only half the bandwidth of the traditional scheme for the same amount of contained information. Toward this end, the four single-sideband (4-SSB) approach for high order modulation is a promising approach for the next-generation communications by applying soft-input soft-output (SISO) equalizer algorithms over orthogonal frequency division multiplexing (OFDM). However, OFDM is challenging for realizing the feasible 5G communications, compared to the emerging techniques, e.g., non-orthogonal multiple access (NOMA), orthogonal multiple access (OMA) or multiple-input multiple-output (MIMO). Since the 4-SSB is an orthogonal modulation which was successfully applied over the traditional OFDM, in this article, we propose a novel 4-SSB modulation scheme over OFDM Guard Interval (GI) and massive MIMO. Besides the carrier signal, from the receiver side, we also apply the shadow equalizer algorithm in the uncoded and coded environment using turbo codes to achieve the 4-SSB with high efficiency from low complexity and energy consumption for 5G. The evaluation results validate that our system consumes lower energy due to low complexity gained from same number of iterations without the heavy decoding as of the 4-SSB SISO based on the turbo equalizer. In addition, the 4-SSB over the OFDM GI achieves the best performance among the relevant approaches conducted in 4-SSB. The proposal then acts as a practical communication system designed to solve the inter-symbol interference (ISI) induced by additional Hilbert transform in the wireless environment toward fifth generation (5G), given that turbo code is considered as a potential channel coding scheme for 5G radio specification.

## 1. Introduction

The fifth generation (5G) technology is about to be launched soon to match the user demand and various requirements of future wireless communications [1]. Besides the machine type communications for ultra-reliability and low latency, enhancing mobile broadband is considered as a main research topic toward the next-generation communication network for 5G [2]. 

In this context, information-centric network (ICN) is regarded as a promising future Internet design with the key innovative features including in-network caching and name-based forwarding to improve the network efficiency [3]. However, by default, ICN requires caching-enabled routers which consume higher power compared to the traditional host-to-host architecture as analyzed in our previous work [4,5,6]. Also, the original forwarding strategy in ICN, leave copy everywhere (LCE), produces high cache redundancy that discourages ICN feasibility for the real-world deployment [7]. 

Different attempts were then made to realize the new modulation spectrum with high efficiency for next-generation communications. Particularly, orthogonal frequency division multiplexing (OFDM) is used in 4G to increase the modulation capacity and feed multi-user synchronically. For 5G, the modulation is usually conducted in non-orthogonal multiple access (NOMA) and orthogonal multiple access (OMA) to realize the feasible alternative modulations [8]. Another potential research trend in 5G is to improve the OFDM scheme by adjusting its structure to fulfill the requirements of 5G such as guard interval discrete Fourier transform and spectrally-preceded OFDM, namely GI DFT-s-OFDM and SP-OFDM, respectively [2,9].

The four single-sideband (4-SSB) [10,11] is another notable work in OFDM which has an advantage of sending double the amount of similar information using only half of the bandwidth compared to other OFDM modulations. The 4-SSB technology can be extended by using a Hilbert transform which allows the generation of the single-sideband (SSB) from different types of modulation like phase shift keying (PSK), quadrature phase shift keying (QPSK), and quadrature amplitude modulation (QAM). However, to the best of our knowledge, this innovative idea of using the SSB technique has not been applied for 5G communications.

Toward this end, we have investigated how QAM modulation can allow a communication system to improve the data rate to match 5G requirements [12]. Hence, in this research, we aim to increase the communication bandwidth by moving toward the combined 4-SSB signals generated from two symbols of QAM. This proposal then can increase the spectrum efficiency through QAM by applying the novel idea of 4-SSB in multiple-input multiple-output (MIMO). 

The proposal complexity can be minimized by redesigning the receiver side, particularly in the equalizer. Typically, we apply a new algorithm in the 4-SSB system, called shadow equalizing. By simulation, the new design demonstrates the low complexity by removing the process of the decoder and interleaver in iteration loops needed for the turbo equalizer. This process also refers to low energy consumption by ascending hardware component in the receiver side [13]. In our prior research [12], we successfully transmitted 16-QAM and 64-QAM through an additive white Gaussian noise (AWGN) environment and multiple path fading channels. The evaluation results show that the M-QAM 4-SSB OFDM can increase channel capacity and data rate. Thus, SSB modulation is a promising candidate to improve efficiency in the 5G network.

Currently, most studies have applied SSB modulation for fiber optics to enable high-speed data rate and increase capacity [14]. However, they also verify that the intersymbol interference (ISI) is the critical issue in SSB-based modulation for the wireless channel. Along this line, our proposal aims to increase the capacity of SSB by compensating ISI in uncodec and codec system using turbo codes, given that turbo code is considered as a potential channel coding scheme for 5G radio specification within Verizon’s 5G Technology Forum (V5GTF) [15]. 

Overall, in this paper, we propose a new modulation communication scheme using 4-SSB over modulated techniques including OFDM and MIMO to improve the channel capacity and data rate of the communication systems towards 5G. To achieve this goal, we develop a shadow minimum mean square error (MMSE) equalizer for the 4-SSB modulated systems to diminish the system complexity. We then verify the system design under both uncoded and coded environments using turbo codes following the 5G specification document as a practical contribution toward feasible massive MIMO (m-MIMO) system implementation. Typically, we investigate and propose an enhanced mobile broadband scheme by addressing the challenging requirements in 5G through potential architectural design including the application in m-MIMO and new modulation spectrum for highly efficient communications with low cost and low complexity at the same time.

## 2. Related Work

The single-sideband (SSB) is sensitive in wireless communications due to the high ISI caused by the wireless environment. Thanks to the Hilbert transformation, it is feasible to generate the SSB signal from different types of modulation, e.g., PSK, QPSK, and QAM. However, for simplicity, the previous SSB-based researches are mainly applied in QPSK for increasing channel capacity. The result showed the successfully transmitted signal which compensates the ISI by using the turbo equalizer algorithm called widely MMSE estimation. The limitation of this approach is that the turbo equalizer is only used for QPSK then performance and feasibility are degraded in multipath fading channel because of the ISI increase [16].

A notable research investigated the increased capacity by combining four single-sideband (4-SSB) QPSK [10]. This technology can double the amount of information by sending two QPSK symbols compared to the traditional SSB QPSK. Thus, undoubtedly, it increases the number of required SSB signals using additional Hilbert transform applied to make ISI. To solve this issue, the authors also applied the turbo equalizer to deal with QPSK modulation for the enhanced efficiency.

The prior SSB-based researches are applicable in second generation (2G) and third-generation (3G) wireless technology. Besides, the research of QPSK 4-SSB using OFDM channel proves that the 4-SSB is applicable in fourth generation (4G) [11]. It also can be applied in the multipath fading channel by using the widely linear MMSE equalizer. The extension of this research is applying inter-canceller to improve the 4-SSB bit error rate efficiency. Currently, there is a need to apply the new idea of 4-SSB for the 5G wireless technology, but the existing researches mainly use turbo equalizers in QPSK.

Typically, the research on 4-SSB M-QAM modulation using soft input soft output (SISO) equalizer over OFDM was proposed for increasing high data rate and capacity [12]. The researchers applied a new turbo equalizer algorithm for high order modulation. The evaluation results showed a consistent performance in AWGN and fading channel. However, we need to decrease the receiver complexity without losing orthogonality for the practical applications in 5G, e.g., MIMO system [17].

To do this, the complexity was reduced by applying the shadow area constraints of QAM using multiple feedback successive interference cancellation with shadow area constraints (MF-SIC-SAC) [13]. Shadow equalizer is a type of equalizer which uses shadow area constraint (SAC) to evaluate the quality of decisions and avoid unnecessary multiple feedback procedures for reliable estimations. This algorithm feeds multiple candidates for symbol estimation. However, a reduced symbol candidate’s decision is still required for realizing low energy and complexity in the equalizer.

The guard interval discrete Fourier transform spread OFDM GI DFT-s-OFDM, and spectrally-preceded OFDM SP-OFDM are feasible candidates of OFDM technology to be applied in 5G [2,9]. The evaluation results of 4-SSB OFDM in uncoded environment showed good performance compared to traditional OFDM thanks to the high bandwidth efficiency from the feature of SSB-based modulation. However, decreasing bit error is required for high efficiency of bandwidth usage and efficient equalization algorithm. Also, we apply turbo code with interleaver parameters as defined by V5GTF for the 5G radio specification [15] in 4-SSB. Our proposal then investigates and proposes the system design with high bandwidth efficiency and low complexity which implies the low energy consumption of the communication systems.

## 3. 4-SSB System Design

### 3.1. Introduction to Single-Sideband Modulation

**Figure 1:**
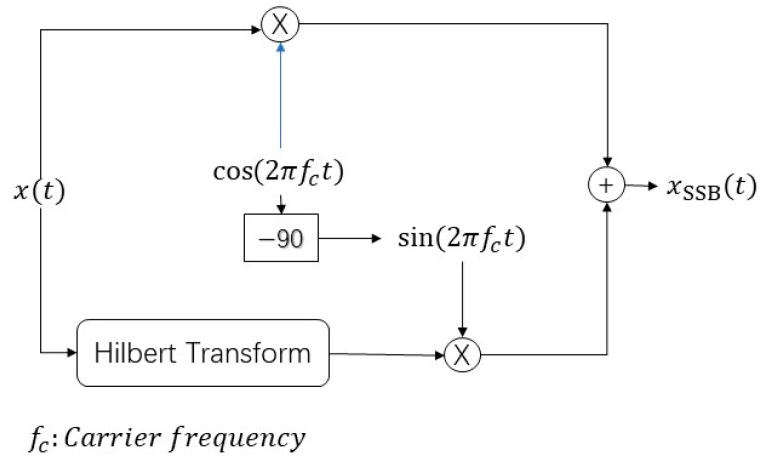
Single-sideband (SSB) generated by the Hilbert transform.

In general, the single-sideband (SSB) [18] is extracted from the double sideband (DSB) which contains two sidebands: the upper sideband (USB) and lower sideband (LSB). Both contain the complete information of the original baseband signal. The SSB modulation only requires half of the signal bandwidth. In Figure 1, without loss of generality, let x(t) be the baseband signal, then the mathematical model of two sidebands USB and LSB in the time domain can be expressed as:

(1) $$x_{\mathrm{USB}}\left(t\right)=\frac{1}{2}\left[x\left(t\right)+j\hat{x}\left(t\right)\right],$$

(2) $$x_{\mathrm{LSB}}\left(t\right)=\frac{1}{2}\left[x\left(t\right)-j\hat{x}\left(t\right)\right],$$

where $\hat{x}\left(t\right)$ is unknown and *j* is a complex value equal to $\sqrt{-1}$.

Then, to identify $\hat{x}\left(t\right)$, we apply the Fourier transform in one sideband of SSB with the opposite sign as follows:

(3) $$X_{\mathrm{USB}}\left(f\right)=X\left(f\right)U\left(f\right)\;=\frac{1}{2}X\left(f\right)\left[1+\mathrm{sgn}\left(f\right)\right]\;=\frac{1}{2}X\left(f\right)+\frac{1}{2}X\left(f\right)\mathrm{sgn}\left(f\right),$$

where U(f) is a step function in the frequency domain.

We observe the Fourier transform: $j\hat{x}\left(t\right)↔X\left(f\right)\mathrm{sgn}\left(f\right)$. Therefore:

(4) $$\hat{X}\left(f\right)=-jX\left(f\right)\mathrm{sgn}\left(f\right),$$

where sgn(f) denotes a signum function in the frequency domain. 

As the inverse Fourier transform for −jsgn(f) in time domain is $\frac{1}{\pi t}$, (4) in time domain is equivalent to:

(5) $$\hat{x}\left(t\right)=x\left(t\right)\;*\frac{1}{\pi t}\;,$$

where ∗ denotes the convolution operation, i.e.:

(6) $$\hat{x}\left(t\right)=\frac{1}{\pi }\int_{-\infty }^{\infty }\frac{x\left(\tau \right)}{t-\tau }d\tau .$$

Specifically, we apply the unit step function to define Hilbert impulse response in the time domain by using a Fourier transform. Then, we finalize the Hilbert transform model as shown in Equations (5) and (6).

The SSB signal generated by the Hilbert transform then can be expressed as:

(7) $$x\left(t\right)=\;\frac{1}{2}\;x\left(t\right)cos\left(2\pi f_{c}t\right)\pm \;\frac{1}{2}\hat{x}\left(t\right)sin\left(2\pi f_{c}t\right),$$

where $f_{c}$ is the carrier frequency and ± denotes the sign of two sidebands: the minus sign is for USB and the positive sign is for LSB. Also, $\hat{x}\left(t\right)$ is a Hilbert transform of signal x(t) and the second term of the right side shows the output generated by Hilbert transform (Figure 1) where x(t) can be represented in the complex form as: $x\left(t\right)=\;x\left(t\right)+j\hat{x}\left(t\right)$.

### 3.2. Four Single-Sideband Modulation

This section describes 4-SSB modulation model using M-QAM [10,11]. The proposed model uses the ideal Hilbert transform of the baseband signal *x*. The analytical mathematical model of the upper sideband generated from baseband signal *x* denoted as $x_{\mathrm{USB}}$, can be expressed as:

(8) $$x_{\mathrm{USB}}\left(t\right)=\frac{1}{2}\left[x\left(t\right)+j\hat{x}\left(t\right)\right].$$

Since the lower sideband is similar to the analytical value of upper sideband but with a different sign, we can define $x_{\mathrm{LSB}}$ it in the same way as of the upper side:

(9) $$x_{\mathrm{LSB}}\left(t\right)=\frac{1}{2}\left[x\left(t\right)-j\hat{x}\left(t\right)\right].$$

**Figure 2:**
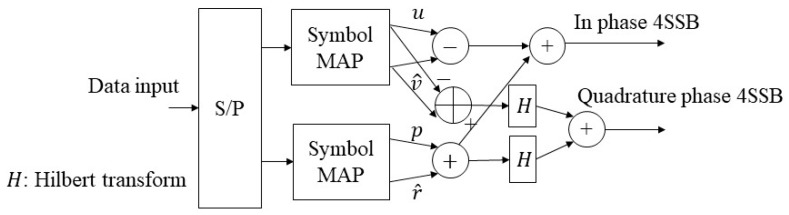
The Four Single-sideband (4-SSB) Modulation Model.

These two expressions lay down the concept of SSB modulation which was used for generating 4-SSB modulation in our prior work [10]. Figure 2 shows the model configuration of 4-SSB in which the 4-SSB modulating expression can be illustrated by considering four independent real discrete sequences, denoted by *u, v, p,* and *r*. The Hilbert transforms of original discrete signals for the designed modulation system are also described in Figure 2. 

Specifically, the combined signal is transformed by the Hilbert transform, and in I/Q modulation of two symbols, the imaginary part is decided as the result of the Hilbert transform. The 4-SSB signal is generated by these four signals corresponding to the following equation:

(10) $$S_{4\mathrm{SSB}}=S_{4\mathrm{SSB},I}+jS_{4\mathrm{SSB},Q},$$

where $S_{4\mathrm{SSB},I}$ and $S_{4\mathrm{SSB},Q}$ denote the In phase 4-SSB and quadrature modulation phase 4-SSB, respectively (Figure 2), in which:

(11) $$S_{4\mathrm{SSB},I}=u-\hat{v}+p+\hat{r},$$

(12) $$S_{4\mathrm{SSB},Q}=-\hat{u}-v+\hat{p}-r,$$

where $\hat{u},\;\hat{v},\hat{p}\;\mathrm{and}\;\hat{r}$ denote the Hilbert transforms of original discrete signals. 

From the previous work of 4-SSB-based modulation technique over QPSK, these equations can be applied for two binary phase shift keying (BPSK) signals or two complex modulated signals like QPSK and QAM. As a result, the two symbols $d_{1}$ and $d_{2}$ of the M-QAM can be represented as follows:

(13) $$d_{1}=u+jv,$$

(14) $$d_{2}=p+jr.$$

The 4-SSB signal then occupies the two-complex signal bandwidth as of $d_{1}$ or $d_{2}$ but carries twice amount of information. This means that the 4-SSB 16-QAM has the same information as of 64- QAM or the like for the high-order modulation. 

### 3.3. Four Single-Sideband Demodulation

This section describes the 4-SSB QAM demodulation as defined in [11,19]. The brief process steps can be expressed as follows:

(15) $$u-\hat{v}=s_{4\mathrm{SSB},I}+{\hat{s}}_{4\mathrm{SSB},Q},$$

(16) $$v+\hat{u}=-s_{4\mathrm{SSB},Q}+{\hat{s}}_{4\mathrm{SSB},I},$$

(17) $$p+\hat{r}=s_{4\mathrm{SSB},I}-{\hat{s}}_{4\mathrm{SSB},Q},$$

(18) $$r-\hat{p}=-s_{4\mathrm{SSB},Q}-{\hat{s}}_{4\mathrm{SSB},I},$$

where ${\hat{s}}_{4\mathrm{SSB},i}$ is Hilbert transform of In phase 4-SSB and the ${\hat{s}}_{4\mathrm{SSB},Q}$ is a Hilbert transform of quadrature modulation phase 4-SSB [20].

These equations show the partial de-combination of two transmitted complex signals. The Hilbert transform tap must be equal in transmitter and receiver to ensure the quality of the transmission process. Also, it is impossible to recover two complex signals without the use of 4-SSB M-QAM demodulation. The two complex signals can be represented by the upper sideband and lower sideband before feeding into the shadow equalizer as follows:

(19) $$d_{1,\mathrm{LSB}}=\frac{1}{2}[\left(u-\hat{v}+j\left(\hat{u}+v\right)\right],$$

(20) $$d_{2,\mathrm{USB}}=\frac{1}{2}[\left(p+\hat{r}+j\left(r-\hat{p}\right)\right],$$

where $d_{1,\mathrm{LSB}}$ denotes the lower sideband of 4-SSB and $d_{2,\mathrm{USB}}$ denotes the upper sideband of 4-SSB. Typically, by defining a spectral profile of 4-SSB signal in the similar way as we did in our previous work [19]. From this, we can identify the two symbols of QAM as shown in Equations (13) and (14). After the modulation of 4-SSB, the spectra are joined as one which includes the two symbols of $d_{1}$ and $d_{2}$. This spectrum is defined as LSB which includes $d_{1}$ in the real and imaginary part as shown in Equation (19). In the same way, we can deduce the Equation (20).

## 4. The Proposed OFDM Shadow Equalizer-based Scheme for Multiple-Input Multiple-Output (MIMO) System Using 4-SSB M-QAM

In this section, firstly we introduce an overview of the basic concept of the OFDM system. Then, we present the proposed 4-SSB shadow equalizer-based scheme in MIMO. Additionally, we note that the proposed 4-SSB-based modulation scheme can be applied for Internet of Things (IoT) networks utilizing multiple sensors as the distributed nodes which can collect, process, and transmit data from sampled physical signals to the connected IEEE 802.11 Wi-Fi Access Points. These distributed sensor nodes then realize a new modulated transmitter design over OFDM/MIMO at the physical layer to enable wireless sensor networks (WSNs). Typically, the sensors can be coordinated as the modulated transmitters and can be removed or added to the distributed environment as long as the constructed sub-carriers do not overlap. Also, this kind of modulated IoT sensor-enabled communication scheme can enable the modulated IoT energy saving as energy-efficient transmission schemes is a requirement for the efficient data transmission in IoT networks. Typically, the recent IEEE 802.11ah (Wi-Fi HaLow) is the latest low power wide area network (LPWAN) technique which extends the range of Wi-Fi network for the realization of IoT-based networks. IEEE 802.11ah standard supports both OFDM and MIMO modulations with high data-rate and low energy consumption at the same time. This promising approach using multiple interconnected sensors in a communication medium will be detailed in our upcoming research.

### 4.1. The Concept of Applying 4-SSB into OFDM

Wireless communications have become a hot research topic these days due to their huge demands in terms of throughput, quality of service (QoS) and coverage. Various techniques have been developed to improve the performance of the applicable traditional orthogonal frequency division multiplexing (OFDM). As the OFDM scheme is applied by recent wireless systems [21], this section then is dedicated to giving a brief concept of the OFDM scheme. 

The transmission in OFDM based system is divided into several subsequent bits carried by different overlapped sub-channels. The sub-channel’s bandwidth is designed to make sure each sub-channel has less than the coherent bandwidth of the channel. Consequently, each subchannel will occur in flat fading so that ISI can be minimized.

The process starts by transmitting a bit sequence of information. After being mapped into Gray QAM, the two symbols of M-QAM are combined by 4-SSB modulation process from the integrated profile spectrum. Then 4-SSB M-QAM scheme is divided into N parallel streams. Each symbol stream is modulated to a set of *N* subscribers. An inverse discrete Fourier transform (IDFT) block is employed to transform the sub-stream into time domain signal. We denote the *l*th symbol on the *k*th subcarrier as the time signal $X_{l,4\mathrm{SSB}}\left[k\right],\;\mathrm{where}\;l\in \left[0,\infty \right)\;\mathrm{and}\;k\in \left[0,N-1\right]$:

(21) $$x_{l,4\mathrm{SSB}}\left(t\right)=\;ℜ\left\{\frac{1}{T_{sym}}\overset{\infty }{\underset{l=0}{\sum }}\left[\overset{N-1}{\underset{k=0}{\sum }}X_{l,4\mathrm{SSB}}\left[k\right]e^{j2\pi f_{k}\left(t-lT_{sym}\right)}\right]\right\}.$$

where ℜ is real part, $T_{sym}$ is the time duration of a single OFDM symbol equaled to N$T_{S}$. where $T_{S}$ is the duration of the original symbol $X_{l,4\mathrm{SSB}}\left[k\right]$ in OFDM. $f_{k}$ is the frequency of subcarrier k.

After applying the 4-SSB modulation scheme on two symbols, we can obtain the $x_{4-\mathrm{SSB}}$ signal as shown in Equation (21). In this way, we can transmit the obtained 4-SSB symbols by using OFDM.

The IDFT produces the discrete time-based OFDM signal at $t=lT_{sym}+nT_{s}$ which can be identified as:

(22) $$x_{l,4\mathrm{SBB}}\left[n\right]=\overset{N-1}{\underset{k=0}{\sum }}X_{l,4\mathrm{SSB}}\left[k\right]e^{\frac{j2\pi kn}{N}}\;\;\;for\;n=\;0,1,2,\ldots ,N-1.$$

In the receiver side, the receiving continuous time OFDM symbol can be expressed by the following equation:

(23) $$y_{l,4\mathrm{SSB}}\left(t\right)=\;\overset{N-1}{\underset{k=0}{\sum }}X_{l,4\mathrm{SSB}\;}\left[k\right]e^{j2\pi f_{k}\left(t-lT_{sym}\right)},\;lT_{sym}<t<lT_{sym}+nT_{s}$$

$X_{l,4SSB}$ can be demodulated by using orthogonal sub-carrier for low complexity where we neglect channel and White Gaussian noise from the expression:

(24) $$\begin{array}{l} Y_{l,4SSB}\left[k\right]=\frac{1}{T_{sym}}\int_{-\infty }^{\infty }y_{l,4SSB}\left(t\right)e^{-j2\pi f_{k}\left(t-lT_{sym}\right)}\;dt\\ \;=\frac{1}{T_{sym}}\int_{-\infty }^{\infty }\left\{\sum_{i=0}^{N-1}X_{l,4SSB\;}\left[i\right]e^{j2\pi f_{i}\left(t-lT_{sym}\right)}\right\}e^{-j2\pi f_{k}\left(t-lT_{sym}\right)}\;dt\\ \;=\sum_{i=0}^{N-1}X_{l,4\mathrm{SSB}\;}\left[i\right]\left\{\frac{1}{T_{sym}}\int_{0}^{T_{sym}}e^{j2\pi (f_{i}-f_{k})\left(t-lT_{sym}\right)}dt\;\right\}=X_{l,4\mathrm{SSB}}\left[k\right] \end{array}$$

Then, the sampled signal is received by the OFDM symbol $y_{l,4\mathrm{SSB}}\left(l\right)$ at $t=lT_{sym}+nT_{s}$ with discrete time ${\left\{y_{l,4\mathrm{SSB}}\left(n\right)\right\}}_{n=0}^{N-1}$. Using fast Fourier transform (FFT) for the discrete time equation, we have:

(25) $$\begin{array}{l} Y_{l,4\mathrm{SSB}}\left[k\right]=\sum_{i=0}^{N-1}y_{l,4\mathrm{SSB}}\left(n\right)e^{\frac{-j2\pi kn}{N}}=\sum_{k=0}^{N-1}\left\{\frac{1}{N}\sum_{i=0}^{N-1}X_{l,4\mathrm{SSB}\;}\left[i\right]e^{\frac{j2\pi in}{N}}\right\}\;e^{\frac{-j2\pi kn}{N}}\\ \;=\frac{1}{N}\sum_{k=0}^{N-1}\sum_{i=0}^{N-1}X_{l,4\mathrm{SSB}\;}\left[i\right]e^{\frac{j2\pi \left(i-k\right)n}{N}} \end{array}$$

### 4.2. The Proposed New Scheme of 4-SSB with Low Complexity Equalizer for Compensating ISI

The quality of service (QoS) is a key network metric of a 5G wireless system, i.e., the successful transmission scheme which recovers the reserved signal in the receiver is important for measuring performance quality in the wireless environment. However, most of the current researches have focused on enabling the receiver to compensate for the effect of noise on the wireless channel. In particularly, the inter-symbol interference (ISI) is used to identify and compensate the symbol interference between orthogonal symbols in the receiver side for 4-SSB by utilizing the Hilbert transform. ISI is produced by the channel impulse response duration less than the time symbol modulation. In this context, channel coding, MIMO technology, and channel equalization are mainly designed for improving QoS performance in wireless environments.

In this research, we propose a new transmission scheme of 4-SSB with low equalizer complexity and low energy consumption. Before presenting our architectural design, we briefly present the types of equalizer category [22]: First type of equalizer is the zero-forcing (ZF) channel equalization. The main idea of forcing equalization is that the inversed channel impulse responds $H^{-1}$ is used in the equalizer of the receiver side to equalize the original channel impulse response H. However, the disadvantage of this technology is increased in the implication noise, especially when the channel impulse response is very small which results in high attenuation. To minimize this effect, the MMSE equalizer is applied to improve the zero forcing equalizer performance. 

The second type of equalizer is the decision feedback channel equalizer. This method of equalization includes the first equalization of zero forcing with first symbol entry which should be known to reduce the order of complexity. An advancement for providing high performance is to add coding channel by applying the convolution encoder and interleaving in transmission and vice versa in a receiver to minimize the error propagation in the receiving channel. This technique is called turbo equalizer, which is mainly designed to compensate the ISI by using an iterative algorithm. The system uses a decoding and equalizing scheme to feed the prior information and, in this way, the equalizer can be used for compensating ISI.

In our prior work, turbo equalization was applied to realize the high order M-QAM 4-SSB [12] by designing the equalizer for the soft input soft output (SISO) MMSE equalizer. The result showed that we can successfully transmit M-QAM through a variety of channel environments such as AWGN and multipath fading channel. The presented article is then dedicated to decreasing the complexity of the equalizer to be applied in the MIMO scheme.

The third type of equalization aims to decrease the complexity of the equalization which can be considered as an iterative MMSE algorithm corresponding to the principle of the shadow area. This equalization is designed to make the decision of the first symbol by estimating whether the signal is strong or weak. If it is weak, we will cancel the estimation when it is considered not close to the estimated value of the original signal to prevent the error propagation. 

Then, many researches examine how to make optimal estimation with low complexity. For example, the maximum likelihood (ML) algorithm is applied for the sphere decoder (SD) [22] and lattice code [23]. However, these two algorithms produce high complexity for high order modulation when the channel is not in good condition where the signal to noise ratio (SNR) is low. For MIMO, the optimum maximum likelihood detection (MLD) shows a good performance by increasing the number of antennas, users and modulation level. In the other hand, a novel vertical algorithm, called Vertical Bell labs layer space-time V-BLAST [24], is used in interference cancellation (IC) to achieve a better performance than the prior algorithms in which the detector made by sphere interference cancellation (SIC) is affected by error propagation. 

In this article, we propose the new scheme of M-QAM 4-SSB OFDM multiple feedback which is used for interference cancellation with shadow area constraints (MF-SIC-SAC). The proposal is divided into four sub-sections in the 4-SSB scheme to enable low complexity and save energy for a wide range of applicable scenarios in 5G. The research is organized as follows:

(1) M-QAM 4-SSB uncoded using shadow equalizer and its performance in terms of bit error rate (BER) over the relevant schemes including MIMO and OFDM GI (Guard Interval). Besides, we apply the proposal in codec environment using a turbo coding scheme to verify the feasibility for modulation implementation toward 5G communications.
(2) The proposed M-QAM 4-SSB over OFDM scheme using Shadow equalizer and its performance including complexity evaluation compared to previous work of M-QAM 4-SSB over OFDM using SISO equalizer.
(3) Comparing the proposed scheme to the related OFDM scheme in 5G.
(4) Applying the proposal into massive MIMO and demonstrating the system efficiency over equivalent systems in MIMO.

### 4.3. The Concept of Application of 4-SSB into OFDM M-QAM 4-SSB Uncoded System Using the Shadow Equalizer

In this section, we apply the multiple feedback success interference cancellation with shadow area constraints (MF-SIC-SAC). This algorithm is utilized to address the error propagation in compensating the ISI and make feedback decision using the SIC technique to test the SNR symbol as feedback. We then propose the new scheme of M-QAM 4-SSB OFDM with MF-SIC-SAC which declines the first symbol estimation using the third aforementioned equalizer type by decreasing the number of feedback symbols. The correct constellation is still allocated in the remaining feedback symbol [13]. 

The selective algorithm is then optimized from the set of candidate’s symbol feedbacks. This optimization algorithm is performed by selecting only one branch in the lattice tree. Consequently, this approach realizes a smart interference canceller, which is different from the hard decision of SIC or sphere decoder by searching in the optimized branch of the lattice tree to prevent from growing complexity. The shadow area constraint is also introduced to decide whether the symbol estimation is reliable or not. The feedback output is a reliable symbol whereas the non-reliable symbol will be replaced by the concentrated symbol producing by SAC.

#### 4.3.1. System Model

**Figure 3:**
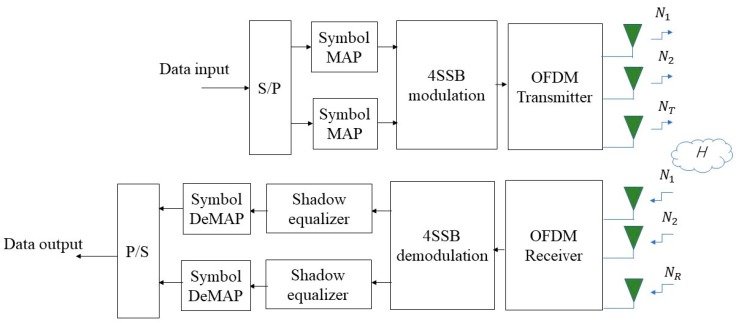
The proposed 4-SSB multiple-input multiple-output (MIMO) system.

The proposed system model is depicted in Figure 3. The system has two major devices in a typical communication system: transmitter and receiver. Suppose that the traditional MIMO system has $N_{T}$ transmitter antennas and $N_{R}$ receiver antennas and the number of receiver antennas generate more than that of transmitter antennas i.e., $N_{R}>\;N_{T}$. Firstly, the transmission scheme is fed by the independent data binary value {1,0} with equal probability. Each binary in the uncodec system then will be mapped using two parallel QAM Gray codec symbols. For M-QAM signals, the complex value is represented in the real and imaginary part of the symbol which takes value in the general set of two M-QAM proceeded by 4-SSB modulatio $\left\{\pm 1,\pm 3,\ldots ,\pm \left(\sqrt{M}-1\right)\right\}$ to produce one profile called 4-SSB M-QAM with the same bandwidth as of two symbols of QAM. 

To realize a feasible approach in MIMO, we translate the system transmitter into $N_{T}\;\times \;1$ vector $x_{4-SSB,n}\left[i\right]={\left[x_{4-SSB,1}x_{4-SSB,2}\ldots x_{4-SSB,N_{T}}\right]}^{T}$. This sequence of symbols is transmitted over a flat fading channel. The 4-SSB M-QAM is first demodulated by the OFDM modulation then follows 4-SSB demodulation process and sampled in the receiver with $N_{R}$ antennas. The received signal after demodulation process is collected in $N_{R}\;\times \;1$ $r\left[i\right]\;=\;{\left[r_{1}\left[i\right],\;r_{2}\left[i\right],\ldots \;,r_{N_{r}}\left[i\right]\right]}^{T}$ and can be expressed as:

(26) $$r\left[i\right]=\;Hx_{4-\mathrm{SSB},n}\left[i\right]+\;v\left[i\right].$$

After the signal is sampled. The signal can be represented in the discrete-time equivalent channel which is affected by additive, zero-mean, circularly symmetric white Gaussian noise v[i] with total variance $\sigma_{v}^{2}$. The receiver signal mathematical model is:

(27) $$r_{n}=\;\sum_{l=0}^{L}h_{l}x_{4-\mathrm{SSB},n-1}\;+\;v_{n},$$

where the set {$h_{l}$} denotes the *L*+1 coefficient of the discrete time equivalent model. In MIMO, the element $h_{nR,nT}$ of the $N_{R\;}\;\times \;N_{T}$ channel matrix H denotes a complex channel gained from the $n_{T}^{th}$ transmitted antenna and $n_{R}^{th}$ with receiver antenna. For simplicity, we consider the *H* complexity corresponds to the optimized ordering. The arrangement is denoted as norm function $\left\|H_{1}\|,\|H_{2}\|,\ldots ,\|H_{N_{T}}\right\|$ where $\left(H_{n}\right)$ represents the *n*th column of *H*.

#### 4.3.2. The Proposed 4-SSB M-QAM MF-SIC-SAC Scheme Design

The proposed system consists of three algorithmic categories. We first introduce the general SIC scheme. Then we explain the branch of MF-SIC. The MF structure requires additional computational complexity. However, shadow concentration is designed to decrease the complexity of the system. The process is made by design of reliable and non-reliable symbol [13].

In general, the conventional SIC algorithm is used in the MMSE filter as $N_{R\;}\times \;1$ filter vectors corresponding to all the antennas. The function of MMSE is to estimate the symbol by making the candidate symbol as null or successful cancellation for perfect optimized detection. The stream of the antenna is denotes as ${\hat{d}}_{i,SSB,n}\left[i\right]={\left[{\hat{d}}_{i,SSB,1}{\hat{d}}_{i,SSB,2}\ldots {\hat{d}}_{i,SSB,N_{R}}\right]}^{T}$ where the receiver $\hat{d}$ is an estimated symbol of 4-SSB. The SIC algorithm of the estimated symbol is expressed as follows:

(28) $${\hat{d}}_{i,SSB,n}\left[i\right]=Q\left(w_{n}^{H}\;{\overset{˘}{d}}_{i,SSB,n}\left[i\right]\right),$$

where the Q(.) function denotes the hard slice function of the receiver symbol which is summarized in the following conditional equation:

(29) $${\overset{˘}{d}}_{i,SSB,n}\left[i\right]=d_{i,SSB,n}\left[i\right],n=1,\;{\overset{˘}{d}}_{i,SSB,n}\left[i\right]=\;d_{i,SSB,n}\left[i\right]-\;\overset{n-1}{\underset{k=1}{\sum }}{\left(H\right)}_{k}{\overset{˘}{d}}_{k}\left[i\right],\;\;\;\;\;\;\;\;\;\;\;\;n\leq \;2,\;w_{n}=\;{\left({\bar{H}}_{n}{\bar{H}}_{n}^{H}+\;\sigma^{2}I\right)}^{-1}{\left(H\right)}_{n}\;\;$$

where ${\bar{H}}_{n}^{H}$ denotes the represented matrix with columns $n,\;n+1,\ldots \;N_{T}$ of *H*. ${\overset{˘}{d}}_{n}$ is the receiving symbol of the 4-SSB post-demodulation process. This symbol is obtained after the constellation of the previously detected (n−1) symbols.

#### 4.3.3. The MF-SIC in 4-SSB Scheme

**Figure 4:**
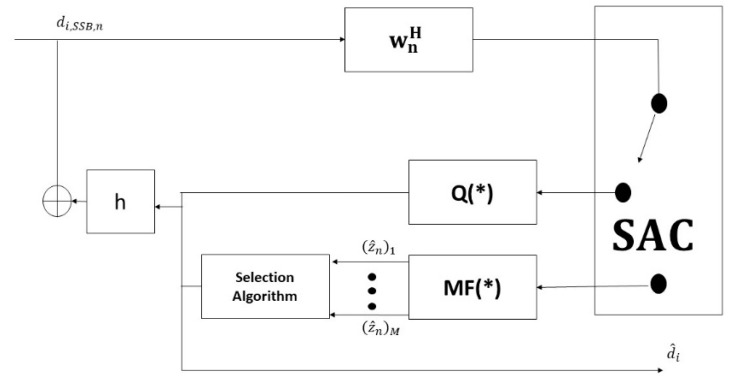
The shadow equalizer in the 4-SSB MIMO System.

The proposal of MF-SIC is 4-SSB in shown in Figure 4. 

The two symbols enter the MMSE to decrease error estimation so that the candidates of symbol detection can be reflected. Without the loss of generality, we represent the process through the pseudocode algorithm of MF-SIC-SAC [13] as in Algorithm 1. The system starts by detecting each data stream ${\hat{d}}_{1},{\hat{d}}_{2},\ldots ,\;{\hat{d}}_{N_{T}}$ which can be obtained in each stage. The constellation is located within the shadow. The algorithm of soft decision ${\tilde{d}}_{n}\left[i\right]\;=\;w_{n}^{H}\;d\left[i\right]$ is applied to check the decision of symbol is reliable or non-reliable as follows [13]:

(1) Reliable Decision: if the algorithm of SAD considers that the symbol ${\hat{d}}_{n}\left[i\right]$ is reliable, the function of hard slice decision is used for both data stream for the hard decision as well as the cancellation function for the next symbol. The conventional in SIC algorithm will match hard slice decision as follows:
  (30) $${\hat{d}}_{n}\left[i\right]\;=\;Q\left({\tilde{d}}_{n}\left[i\right]\right)$$
(2) The symbol is decided as non-reliable if the previous exhaustive decision is non-reliable. Then several candidate’s vectors are generated, and the aforementioned conventional SIC will decide the candidate’s symbol as minimal as possible for saving energy saving. Algorithm 1 selects which candidate symbol is the nearest one to the original symbol.

**Algorithm 1.** The Algorithm of MF-SIC-SAC in 4-SSB domain.
**   Initialization of MMSE filter:**

**1**
 $w_{n}=\;{\left({\bar{H}}_{n}{\bar{H}}_{n}^{H}\;+\;\sigma^{2}I\right)}^{-1}{\left(H\right)}_{n},\;n=\;1,\ldots ,N_{T}$
**2**
  **for**
n= 1 to $N_{T}$
**do**
**3**
      ${\tilde{d}}_{n}=\;w_{n}^{H}d_{n}$
**4**
      **If**
${\tilde{d}}_{n}\in$ Shadow Area 
**5**
          ${\tilde{z}}_{n}=\;{\left[\;c_{1},\;c_{2},\;\ldots ,\;c_{M}\right]}^{T}$
**6**
          **for**
m=1 to M
**do**
**7**
              **for**
j= n to $N_{T}$
**do**
**8**
                  ${\hat{d}}_{j,m}=\;{\overset{ˇ}{d}}_{n}\;-\;{\left(H\right)}_{n}\;{\left({\hat{z}}_{n}\right)}_{m}$
**9**
                      $-\sum_{p=\;n+1}^{j-1}{\left(H\right)}_{p}b_{p,m}$
**10**
                  $b_{j,m}=\;Q\left(w_{j}^{H}{\hat{d}}_{j,m}\right)$
**11**
              **end for**
**12**
          **end for**
**13**
          $b_{m}=\;{\left[b_{1,m},\ldots ,b_{N_{T,},m}\right]}^{T}$
**14**
          $m_{opt\;}=\arg\min{\;}_{1\leq m\leq M}$
$\|d_{i,SSB,n}-Hb_{m}{\|}^{2}$
**15**
          ${\hat{d}}_{n}=\;{\left({\hat{z}}_{n}\right)}_{m_{opt}}\;$
**16**
      **else**
**17**
          ${\hat{d}}_{n}=\;Q\left({\tilde{d}}_{n}\right)$
**18**
      **end if**
**19**
      ${\overset{ˇ}{d}}_{i,SSB,n}=\;d_{i,SSB,n}\;-\;\sum_{k=1}^{n-1}{\left(H\right)}_{k}{\hat{d}}_{k}$
**20**
  **end for**

Particularly, the selection algorithm is used to choose the best candidate symbol in 4-SSB. The procedure has two steps. First, multiple feedback (MF) is generated. As shown in Figure 4, the MF algorithm is applied after SAC makes the non-reliable decision.

To optimize the symbol candidate in the first iteration, we select the nearest *M* constellation points by the soft decision of MMSE filter ${\tilde{d}}_{n}\left[i\right]$. This filter is defined as the function of multiple feedbacks as the following expression:

(31) $$ℒ=\;MF\left({\tilde{d}}_{n}\left[i\right]\right)$$

The constellation of the M-QAM symbol candidates can be represented by the subset of $ℒ={\left[\;c_{1},\;c_{2},\;\ldots ,\;c_{M}\right]}^{T}$ ⊆ A. The nearest M constellation refers to the signal to noise ratio (SNR) as a higher value of SNR implies a smaller value of *M*.

In Algorithm 1, we also introduce a new factor called ${\hat{z}}_{n}\left[i\right]$ which is represented by *M x 1* vectors. The output of MF generates a nearest constellation of symbol candidate represented by:

(32) $${\hat{z}}_{n}\left[i\right]=\;{\left[\;c_{1},\;c_{2},\;\ldots ,\;c_{M}\right]}^{T}$$

This stage is significant to make the decision feedback optimal. The previous step of MF-SIC is to prevent the system from making error propagation.

The second step is for the candidate symbol. In this step, the objective is to select optimal candidates from the symbol ${\hat{z}}_{n}\left[i\right]$. Let $b_{m}$ denote a new vector symbol of the optimal candidates with the range of 1 ≤m≤M.

As each $b_{m}$ represents *m*th candidate in each n to $N_{T}$ stage of processing, the candidate vector symbol is expressed by the following transform matrix:

(33) $$b_{m}\left[i\right]=\;{\left[{\hat{d}}_{n}\left[i\right],\ldots ,{\hat{d}}_{n-1}\left[i\right],\;b_{n,m}\left[i\right],\ldots ,b_{j,m}\left[i\right],\ldots ,b_{N_{T},m}\left[i\right]\right]}^{T}.$$

This vector value is filtered by the MMSE filter described as follows:

(34) $$b_{j,m}=\;Q\left(w_{j}^{H}{\hat{d}}_{j,m}\left[i\right]\right),$$

where ${\hat{d}}_{j,m}$ denotes the cancelled receiver feedback vector in the *m*th stage. The reason of applying MMSE vector is to decrease of the complexity of the system:

(35) $$b_{m}=\;{\left[b_{1,m},\ldots ,b_{N_{T,},m}\right]}^{T}.$$

The algorithm then selects the optimal feedback corresponding to the following constraint:

(36) $$m_{opt\;}=\arg\underset{1\leq m\leq M}{\min}\|d_{i,SSB,n}-Hb_{m}{\left[i]\right\|}^{2},$$

(37) $${\hat{d}}_{n}=\;{\left({\hat{z}}_{n}\right)}_{m_{opt}}.$$

#### 4.3.4. The Constraint of the Shadow of M-QAM

**Figure 5:**
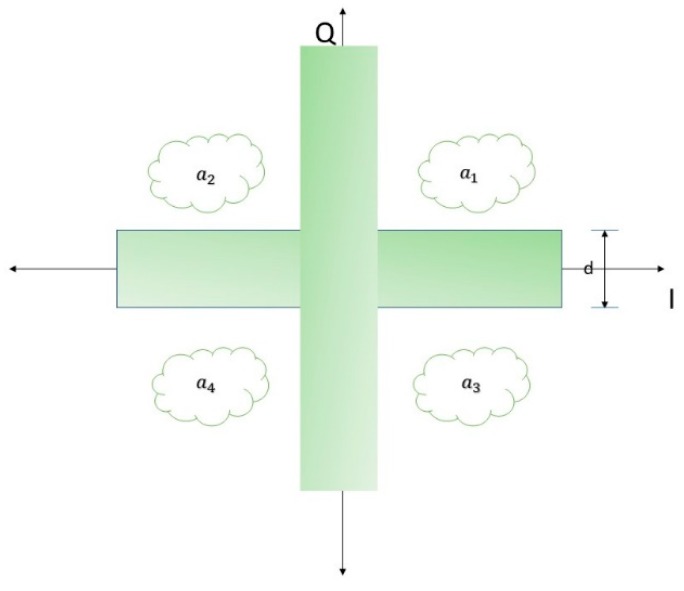
4-QAM Shadow Area Constellation.

Figure 5 represents the shadow constraint in 4-QAM. 

The shadow area has distance d with the original MF algorithm. However, the main difference between the development of decision feedback and the SAC is the feedback representation. Specifically, when the feedback is zero, the error propagation will be increased. SAC avoids null feedback by applying the shadow distance to determine the probability of soft decision as the following equation:

(38) $${\tilde{d}}_{n}\left[i\right]=\;w_{n}^{H}d_{n}\left[i\right].$$

The system avoids the null feedback by generating MF to minimize the error burst. 

In general, QAM constellation can be determined since ${\tilde{d}}_{n}$ refers to reliability rated from shadow distance d [13]:

(39) $$\{\begin{array}{c} \left[ℛ\left[{\tilde{d}}_{n}]-ℛ[a_{K,n}\right]\right]>d/2\\ {}[ℑ[{\tilde{d}}_{n}]-ℑ[a_{K,n}]]>d/2 \end{array}.$$

The high SNR means low complexity as we aim to get the best case for estimating symbol. The remaining part of the equation is $\left[a_{K,n}\right]$ which is the constellation point of M-QAM with the nearest soft decision of ${\overset{ˇ}{d}}_{n}=\;w_{n}^{H}d_{n}\left[i\right]$ in the *n*th layer:

(40) $$a_{K,n\;}=\;arg\;\underset{a_{k}\in \;A}{\min}\|w_{n}^{H}{\overset{ˇ}{d}}_{n}\left[i\right]-\;a_{k}{\|}^{2},\;k=1,\ldots ,C.$$

## 5. Performance Evaluations and Discussion

### 5.1. M-QAM 4-SSB Uncoded System Using Shadow Equalizer Evaluation and Complexity Analysis

#### 5.1.1. Complexity Analysis

The equalization problem can be seen as an iterative MMSE algorithm based on the shadow area principle. As N is the frame length, the first iteration includes the computation of a matrix inversion with the complexity of order $N^{3}$. For the other iterations, the complexity order is about $\left(N-n_{i}\right)N^{2}$ where $n_{i}$ is the number of symbols considered as reliability over the iteration i. Therefore, the total complexity is about $O\left(N^{3}\right)$ dominated by the complexity of the first iteration. 

#### 5.1.2. Performance Evaluation

**Figure 6:**
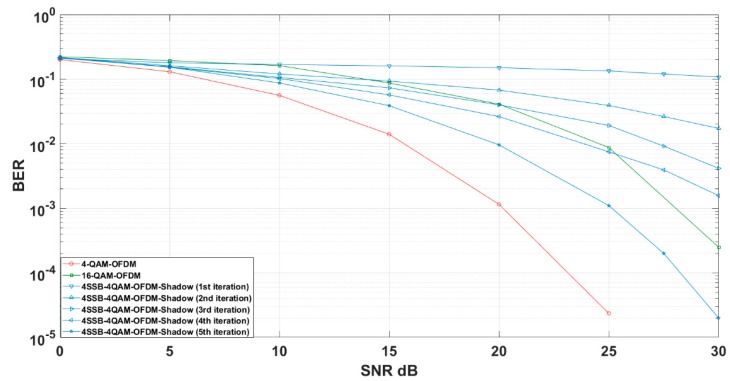
4-QAM 4-SSB Shadow equalization iteration evaluation compared to the relevant modulation schemes.

Figure 6 shows the 4-QAM 4-SSB shadow equalization iteration performance compared to the equivalent scheme in uncoded system.

Overall, the 4-QAM 4-SSB OFDM performance can reach SNR efficiency close to equivalent amount information as of 16-QAM after the fifth iteration as verified in our prior work in 4-SSB [11]. However, the 4-QAM OFDM performs worse than 4-QAM 4-SSB because OFDM scheme carries less amount of bit compared to 4-SSB.

Specifically, the iteration process in 4-QAM 4-SSB shadow initially estimated by ${\overset{ˇ}{d}}_{n}$ since no previous symbol is available with the steady tendency of SNR. In the second iteration, the first estimation is fed as the previous symbol and the (n−1)th iteration is slightly improved compared to the first iteration. In the third iteration, the symbol has good SNR for estimation which is equivalent to 16 QAM-OFDM performance by 20 dB SNR. Similarly, the fourth iteration is equivalent to bit error rate of 16-QAM till 25 dB SNR. In the last iteration, the 4-QAM 4-SSB has the peak efficiency of bit error rate as it can achieve bit error to $10^{-3}$ at 25 dB SNR but the equivalent scheme of 16-QAM OFDM still performs better with the same SNR. The red line demonstrates the performance of constellation of 4-QAM modulation over OFDM.

**Figure 7:**
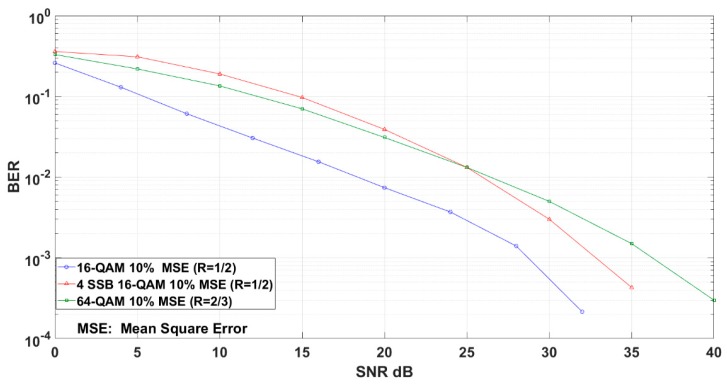
4-QAM 4-SSB with turbo code system using shadow equalization compared to the relevant modulation schemes.

Besides, to verify the efficiency of the proposal for practical deployment, we take turbo code as a classical coding approach for the transport channel, and we fix the Mean Square Error (MSE) value as 10% for the equalizer’s channel estimation used by equalizer to model imperfect equalizer [11]. Specifically, in Figure 7, we compare the BER performance of different modulation schemes in the coded case using turbo code with comparable information data-rates.

We show that the coded 16-QAM with code rate *R = 1/2* outperforms the other modulation schemes; however, it also represents the lowest spectrum efficiency with only two information bits per channel use. In contrast, the 4-SSB 16-QAM (*R = 1/2*) and 64-QAM (*R = 2/3*) modulation schemes represent the same bit rate with four information bits per channel use, and the evaluation result demonstrates that after SNR = 25 dB, the 4-SSB 16-QAM outperforms the 64-QAM by achieving a gain of 3 dB at BER = 10^−3^.

### 5.2. M-QAM 4-SSB Uncoded System Using Shadow Equalizer Evaluation and Complexity in Massive MIMO

The MIMO system is key to modern wireless technology toward 5G. The main goal of MIMO application is to realize the diversity and spatial multiplexing. Diversity enables the system to achieve robust communications through transmission with an independent channel. In the other hand, the spatial multiplexing can save energy consumption by increasing the data rate and efficiently use bandwidth. Specifically, different from single antenna systems in multipath channels, the data rate and capacity of a channel in MIMO are significantly improved by using multiple receive and transmit antennas. Also, MIMO-based wireless communication systems can enable higher data-rate with less required transmission energy under the same throughput requirement compared to the single-input single-output system [25].

**Figure 8:**
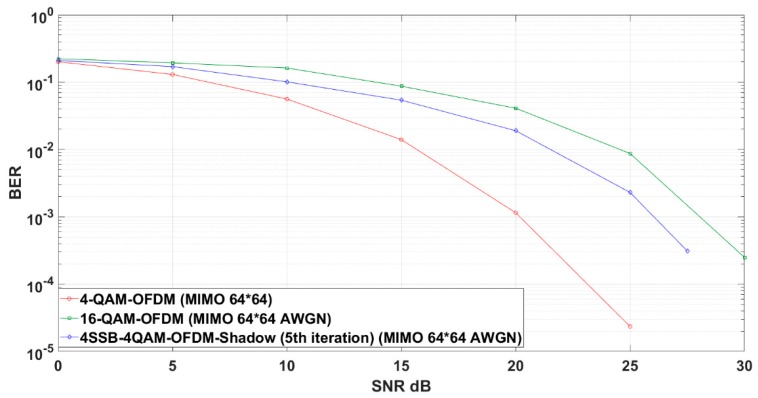
4-QAM 4-SSB shadow in massive MIMO compared to relevant schemes in Additive White Gaussian Noise (AWGN) environment.

In our proposal, the 4-SSB M-QAM performs slightly close to the performance of 16 QAM over the OFDM scheme. However, the 4-SSB M-QAM with shadow equalizer decreases the SNR compared to the previous scenario as we apply both the equalization and massive MIMO. This efficiency is demonstrated after 20 dB SNR. In the other hand, the 4-QAM OFDM has good performance because it carries only two bits (Figure 8). 

**Figure 9:**
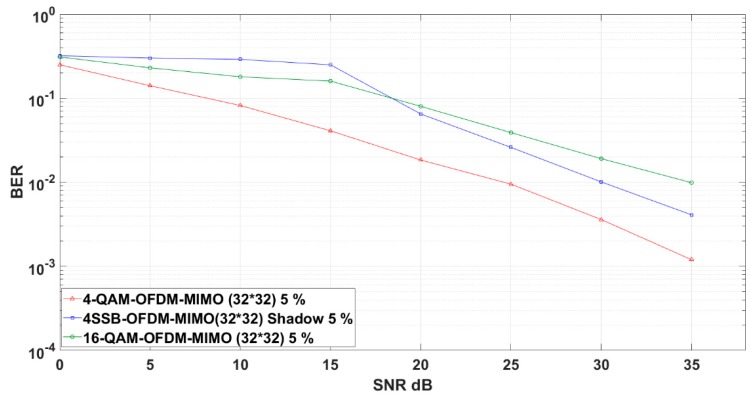
4-QAM 4-SSB shadow in massive MIMO compared to the relevant modulation schemes in Rayleigh Channel environment.

Figure 9 shows the performance evaluations of our 4-SSB-based proposal and other relevant MIMO schemes in which the Rayleigh channel model is applied for the multipath fading channel environment. 

In this channel, the quadrature modulations of 4-QAM and 16-QAM over OFDM applied in 4-SSB are sensitive to the residual phase error, resulting in distances in quadrature-order modulation. The uniform distribution of data sources mapped by QAM Gray coding and the large number of Hilbert transform filter tabs in 4-SSB M-QAM can be approximated as a complex Gaussian distribution. Additionally, the different types of QAM Gray coding of symbols are bit mapped very close to one another. However, we still can obtain sufficient spectral efficiency.

**Figure 10:**
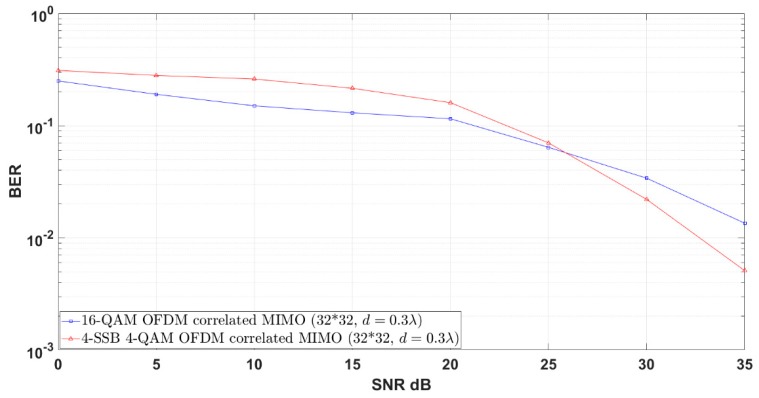
4-QAM 4-SSB shadow in correlated MIMO compared to the relevant modulation schemes in uncoded system under Rayleigh Channel environment.

Next, we consider a more realistic MIMO scenario by showing the impact of the channel correlation. Particularly, we adopt a spatially correlated MIMO fading model using the Kronecker product model [26]. This model assumes the fading statistics of the transmit and receive antennas are independent. The correlation matrices are then computed based on the Bessel distribution correlation and the distance between antennas. In Figure 10, we take the distances between antennas as d = 0.3λ. As the effect of the channel correlation is examined for the uncoded case, we show that the same results can be obtained in the case of non-correlated Rayleigh channel.

**Figure 11:**
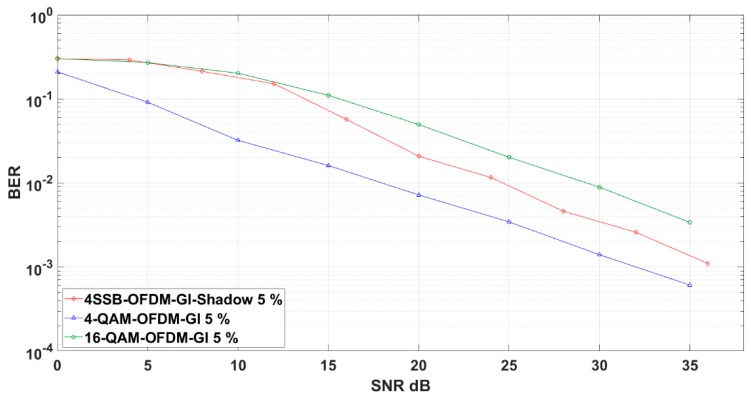
4-QAM 4-SSB shadow applied in orthogonal frequency division multiplexing Guard Interval (OFDM-GI) compared to the relevant modulation schemes in Rayleigh Channel environment.

Finally, we apply 4-SSB using shadow equalizer in OFDM-GI as a new modulation to match the 5G requirements. Specifically, the discrete Fourier transform spread OFDM-GI (DFT-s-OFDM-GI) replaces cyclic prefix (CP) instead of the Guard Interval. In addition, the OFDM-GI can choose the time duration in a flexible way. Typically, this improves the performance of 4-SSB by using additional Hilbert transform for symbol generation. Hence, the Hilbert transform induces delay by 90 degrees, i.e., ISI is increased as well. Figure 11 shows an interesting result by investigating the evaluation history of 4-SSB. 

First, 4 bits in 4-QAM 4-SSB performs close to 2 bits of 4-QAM after 35 dB SNR. The remaining evaluations show that the proposal can reach Shannon boundary in the codec system since it performs well under the harsh condition of the uncoded environment.

## 6. Conclusion and Future Work

In this article, we propose a new modulation which enables high data rate with low complexity for efficient communications toward 5G and beyond. To make the proposal feasible for the 5G communication network, we take into consideration both the receiver and carrier signal to improve the overall system performance. For this goal, we investigate various modulation schemes and identify the best modulation and transmission scheme using 4-SSB by analyzing the system BER performances under different subcarrier settings to verify the feasibility of the system design.

Firstly, we transmit the signal through the uncoded system to minimize the effect of ISI in AWGN and fading multipath channel. The approach of 4-SSB using shadow equalizer can efficiently decrease the complexity of receiver by removing the iterative turbo equalization and decoding process required in a traditional 4-SSB SISO equalizer. In addition, we apply the proposal using turbo coded transport channels with the interleaver parameters as suggested by the V5GTF’s 5G specification regarding multiplexing and channel coding to verify the efficiency and enable the feasibility of the proposal at the same time.

Next, we apply the 4-SSB in massive MIMO to serve the huge demand of the user and enable data rate in the 5G network. The evaluation results demonstrated that the proposed system performs well in the case of 64 × 64 MIMO and 32 × 32 MIMO under the AWGN and Rayleigh channels. We also apply the correlation Rayleigh to realize a realistic scenario in the ideal case toward 5G communications. Typically, we show the improved performance of the proposal over 64-QAM with coding rate 2/3 when using Turbo code for the coded environments. Besides, we achieve the lowest value of SNR in OFDM-GI. 

To enable a complete modulation scheme in various environments toward 5G, handling the Doppler effect and mobility will be our focus points in future work. Also, besides turbo code, we have the plan to conduct the proposed 4-SSB modulation scheme with other promising source coding techniques for 5G implementation, including Polar code with successive cancelation list and linear BLOCK error correction code, and low-density parity-check code (LDPC) with sum-product and linear error correction code. In addition, we will conduct the evaluations using sensors to verify the efficiency of the proposal for the feasible network deployment as a practical fulfillment of massive machine-type communications over MIMO for the next-generation wireless communication systems. Also, as the collected data at all sensor nodes can be interleaved, the effect of fading between the access points and each modulated sensor node can be diminished with increased data rate for various radio applications in IoT sensor-enabled communications.
